# Imaging features and mechanisms of novel coronavirus pneumonia (COVID-19): Study Protocol Clinical Trial (SPIRIT Compliant)

**DOI:** 10.1097/MD.0000000000019900

**Published:** 2020-04-17

**Authors:** Zixing Huang, Shuang Zhao, Lin Xu, Jianxin Chen, Wei Lin, Hanjiang Zeng, Zhixia Chen, Liang Du, Yujun Shi, Na Zhang, Bin Song

**Affiliations:** a Department of Radiology, West China Hospital, Sichuan University, Chengdu; b Department of Radiology, Danzhou Central Hospital, Danzhou; c Department of Radiology, West China-Guangan Hospital, Sichuan University, Guangan; d Department of Radiology, First People's Hospital; e Chinese Evidence-Based Medicine Center, West China Hospital, Sichuan University; f Laboratory of Pathology, West China Hospital, Sichuan University; g Department of Radiology, Public Health Clinical Center, Chengdu, China.

**Keywords:** computer tomography, disease coronavirus disease 2019, multicenter study, prospective observational study, severe acute respiratory syndrome coronavirus 2

## Abstract

**Introduction:**

A novel coronavirus, tentatively designated as 2019 Novel Coronavirus (2019-nCoV), now called severe acute respiratory syndrome coronavirus 2, emerged in Wuhan, China, at the end of 2019 and which continues to expand. On February 11, 2020, the World Health Organization (WHO) named the disease coronavirus disease 2019 (COVID-19). On February 28, WHO increased our assessment of the risk of spread and the risk of impact of COVID-19 to very high at a global level. The COVID-19 poses significant threats to international health.

Computed tomography (CT) has been an important imaging modality in assisting in the diagnosis and management of patients withCOVID-19. Some retrospective imaging studies have reported chest CT findings of COVID-19 in the past 2 months, suggesting that several CT findings may be characteristic. To our knowledge, there has been no prospective multicentre imaging study of COVID-19 to date.

We proposed a hypothesis: There are some specific CT features on Chest CT of COVID-19 patients. And the mechanism of these CT features is explicable based on pathological findings.

**Objective:**

To investigate the specific CT features of COVID-19 and the formation mechanism of these CT features.

**Method:**

This study is a prospective multicenter observational study. We will recruit 100 patients with COVID-19 at 55 hospitals. All patients undergo chest CT examination with the same scan protocol. The distribution and morphology of lesions on chest CT, clinical data will be recorded. A number of patients will be pathologically examined after permission is granted. The data of these three aspects will be analyzed synthetically.

**Discussion:**

This study will help us to identify the chest CT features of COVID-19 and its mechanism.

**Ethics and dissemination:**

This retrospective study was approved by the Biomedical Research Ethics Committee of West China Hospital of Sichuan University (No. 2020–140). Written informed consent will be obtained from all study participants prior to enrollment in the study. To protect privacy of participants, all private information were kept anonymous. The results will be published in a peer-reviewed journal and will be disseminated electronically and in print regardless of results.

## 1 Introduction

A novel coronavirus, tentatively designated as 2019 Novel Coronavirus (2019-nCoV),^[1]^ now called severe acute respiratory syndrome coronavirus 2,^[2]^ emerged in Wuhan, China, at the end of 2019 and which continues to expand. On February 11, 2020, the World Health Organization (WHO) named the disease coronavirus disease 2019 (COVID-19). On February 28, WHO increased our assessment of the risk of spread and the risk of impact of COVID-19 to very high at a global level.^[3]^ As of 24:00 on March 6, the National Health Commission had received 80,651 reports of confirmed cases and 3070 deaths, and in all 55,404 patients had been cured and discharged from hospital. There still remained 22,177 confirmed cases (including 5489 in serious condition) and 502 suspected cases. So far, 672,458 people have been identified as having had close contact with infected patients. 26,730 are now under medical observation.^[4]^ The WHO reported 17,481 laboratory-confirmed cases and 335 deaths in 88 countries /territories/areas as of March 6, out of China.^[5]^ The COVID-19 poses significant threats to international health.

Computed tomography (CT) has a major role in both diagnosis and categorization of COVID-19 on the basis of case definitions issued by the WHO and the treatment guidelines from the National Health Commission of the People's Republic of China. Almost all suspected patients receive chest CT scan. Some retrospective imaging studies have reported chest CT findings of COVID-19 in the past 2 months, suggesting that several CT findings may be characteristic.^[6–18]^ To our knowledge, there has been no prospective multicentre imaging study of COVID-19 to date.

We proposed a hypothesis: There are some specific CT features on Chest CT of COVID-19 patients. And the mechanism of these CT features is explicable based on pathological findings.

## 2 Participants and methods

### 2.1 Study aims

The aim of this study is to investigate the specific CT features of COVID-19 and the formation mechanism of these CT features.

### 2.2 Study design/setting

**Figure 1:**
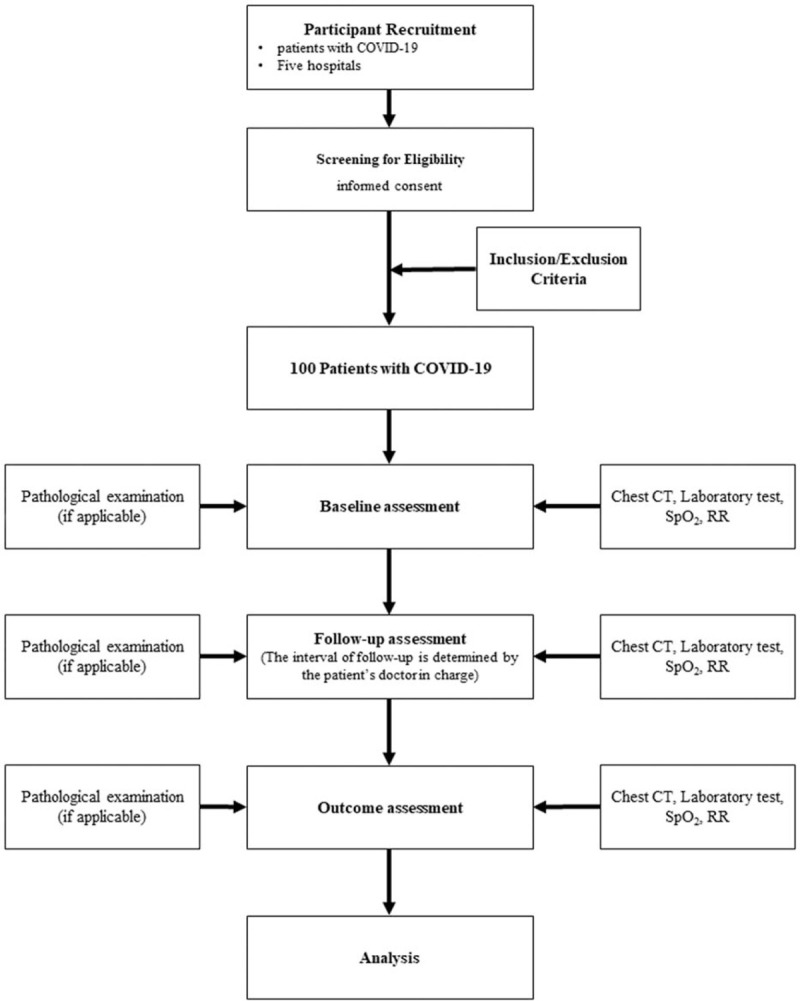
A flowchart of the study. COVID-19 = disease coronavirus disease 2019, CT = computer tomography, RR = respiratory rate, SpO2 = percutaneous oxygen saturation.

This study is a prospective multicenter observational study. The patients will consecutively be enrolled from March 15 to May 15, 2020 in 55 tertiary hospitals, including the Sichuan University West China Hospital, Danzhou Central Hospital, Chengdu First People's Hospital and Chengdu Public Health Clinical Center. All the patients will be hospitalized and have been laboratory confirmed COVID-19. The clinical outcomes will be monitored up to September 15, 2020, the final date of follow-up (Fig. 1).

### 2.3 Study registration

This clinical trial has been registered on the Chinese Clinical Trial Registry (www.chictr.org.cn), and the registration number is ChiCTR2000029764.

### 2.4 Eligibility criteria

#### 2.4.1 Inclusion criteria

We will include patients who are eligible according to the following criteria:

1. The patient will be confirmed to be COVID-19 by laboratory examination.
2. Undergo chest CT scan during the treatment and at least 1 chest CT scan during follow-up.
3. Written informed consent to participate in the study.
4. Compliance with the study regulations.

#### 2.4.2 Exclusion criteria

1. CT image quality does not meet the analysis criteria.
2. Pregnant and lactating women.

### 2.5 Recruitment

We will recruit 100 patients with COVID-19 at 5 hospitals. The researcher will explain the aim of this study and the details of the procedures and will obtain informed consent from potential subjects prior to the collection of information. Participants will be free to withdraw at any time during the study, and this will not affect their clinical treatment.

### 2.6 Intervention

This is an observational study. There are no interventions in this study. The researchers also do not influence the doctors who actually write the diagnosis reports.

### 2.7 CT procedure

#### 2.7.1 CT scanning protocol

The CT scanning protocol of these 5 hospitals adopted the same parameters: tube voltage, 120 kV; tube current, 110mAs (automatic adjustment); rotation time, 0.5 second; section thickness, 0.75 mm; collimation, 0.6 mm; pitch, 1; matrix, 512 × 512; and inspiration breath hold. Reconstruction was performed with a bone algorithm with a thickness of 1 mm and an interval of 1 mm. All scans will be obtained with the patient in the supine position during end-inspiration without intravenous contrast material. The following windows will be used: a mediastinal window with a window width of 350 HU and a window level of 40 HU and a lung window with a width of 1800 HU and a level of 400 HU.

#### 2.7.2 CT features analysis

All CT images will be reviewed by 2 radiologists with 10 years of experience each (S.Z. and W.D.) independently, and final decisions will be reached by consensus. For disagreement between the 2 primary radiologist interpretations, a third radiologist with 15 years of experience (Z.H.) adjudicated a final decision.

The distribution of lung abnormalities will be recorded as:

1. Left, right, or bilateral lung;
2. Predominantly subpleural, hilar, and random;
3. Predominantly superior, inferior, and random;
4. Predominantly anterior, posterior, and random;
5. Presented as a solitary lesion. Numbers of involved lobes and segments of lungs were recorded also.

The CT findings will be described using internationally standard nomenclature defined by the Fleischner Society glossary, including ground glass opacity, consolidation, crazy-paving pattern, bronchiolectasis, interlobular septal thickening, and lymphadenopathy. Other abnormalities will be noted.

### 2.8 Clinical data collection

All the clinical data on epidemiology, signs and symptoms, underlying comorbidities, and laboratory results will be extracted from electronic medical records and checked. The real-time reverse transcriptase polymerase chain-reaction test will be performed. The date of disease onset will be defined as the day when the symptom noticed.

### 2.9 Pathologic procedure

If the patient agrees, we will conduct biopsy pathology. If the deceased signed the body donation while alive, we can perform autopsy to obtain the pathological results.

### 2.10 Outcome measure

The primary outcome of this study is to reveal the characteristic imaging features of COVID-19. The secondary outcome is to reveal the imaging features at different pathological stages.

### 2.11 Data collection and management

Data will be processed anonymously, omitting the information that can identify the participant's individual identity. Strict safety and confidentiality measures will be established in the archives of clinical trial institutions.

### 2.12 Sample size calculation

We calculated the number of patients needed considering the rates of major imaging findings in several published retrospective imaging studies of COVID-19; the estimated sample size is 80 participants according to Lehr's formula. We will recruit 100 patients considering the dropout rate of 20%.

### 2.13 Statistical analysis

For variables with a normal distribution, the independent Student's t test was used. For abnormal distributional variables, the Mann–Whitney *U* test was used. The frequency of CT signs was expressed as the number (percentage) of occurrences and was compared for cases of early- versus advanced-phase disease using the chi-square test or the Fisher exact test. The variables included in the analysis will be subject to single-factor logistic regression, and all variables with significant characteristics will be selected for binary or multiple logistic regression analysis to obtain independent predictors within the group. Finally, the independent predictors in the 2 groups were combined into binary logistic regression analysis to obtain the independent predictors in all variables. For all binary logistic regression analyses, odds ratio and 95% confidence intervals of independent predictors were calculated and model function equations, sensitivity, specificity, and area under the curve were given. Backward stepwise selection was utilized based on the Akaike information criterion.

All statistical analyses were performed with SPSS 20.0 (IBM, Armonk, NY, USA). A 2-tailed *P* value lower than .05 was considered statistically significant.

### 2.14 Ethics and dissemination

This retrospective study was approved by the Biomedical Research Ethics Committee of West China Hospital of Sichuan University (No. 2020–140). Written informed consent will be obtained from all study participants before enrollment in the study. To protect privacy of participants, all private information were kept anonymous. The results will be published in a peer-reviewed journal and will be disseminated electronically and in print regardless of results.

## 3 Discussion

The CT features of COVID-19, reported by these retrospective chest CT studies, are variable, including both relative specificity and nonspecific. We need to know more about this novel highly contagious viral pneumonia: the correlation of CT findings with clinical severity and progression, the predictive value of baseline CT or time changes in disease outcomes, and the correlation of covid-19 with sequelae of acute lung injury remain to be investigated. We hope our research will reveal these issues.

The main limitation of our study is that we could not estimate how many patients would be willing to undergo biopsy and how many families would be willing to donate bodies.

## Author contributions

**Conceptualization:** Zixing Huang, Shuang Zhao, Bin Song.

**Data curation:** Shuang Zhao, Na Zhang, Lin Xu, Jianxin Chen, Wei Lin.

**Formal analysis:** Zixing Huang, Shuang Zhao, Yujun Shi.

**Funding acquisition:** Liang Du.

**Investigation:** Shuang Zhao, Lin Xu, Jianxin Chen, Wei Lin, Hanjiang Zeng, Zhixia Chen, Liang Du, Yujun Shi, Na Zhang.

**Methodology:** Zixing Huang, Liang Du.

**Resources:** Bin Song, Na Zhang, Lin Xu, Jianxin Chen, Wei Lin.

**Supervision:** Bin Song, Na Zhang.

**Writing – original draft:** Zixing Huang, Shuang Zhao.

**Writing – review & editing:** Bin Song.
